# Incidence and risk factors of active tuberculosis among older individuals with latent tuberculosis infection: a cohort study in two high-epidemic sites in eastern China

**DOI:** 10.3389/fcimb.2024.1332211

**Published:** 2024-04-29

**Authors:** Ping Zhu, Xiaogang Hao, Wei Wang, Wei Wang, Bingjun Xu, Bingdong Zhan, Chunfu Fang, Yating Zhang, Yu Gao, Bin Chen

**Affiliations:** ^1^ Department of Tuberculosis Control and Prevention, Quzhou Center for Disease Control and Prevention, Quzhou, Zhejiang, China; ^2^ Department of Tuberculosis Control and Prevention, Zhejiang Provincial Center for Disease Control and Prevention, Hangzhou, Zhejiang, China; ^3^ Zhejiang University of Traditional Chinese Medicine, Hangzhou, Zhejiang, China

**Keywords:** tuberculosis, latent tuberculosis infection, interferon-gamma release assay, older adults, chest X-ray

## Abstract

**Background:**

The influencing factors of the process from latent tuberculosis infection (LTBI) to the onset of active tuberculosis (TB) remain unknown among different population groups, especially among older individuals in high-incidence areas. This study aimed to investigate the development of active TB among older adults with LTBI and identify groups in greatest need of improved prevention and control strategies for TB.

**Methods:**

In 2021, we implemented an investigation among older individuals (≥ 65 years old) in two towns in Zhejiang Province with the highest incidence of TB. All participants underwent assessment using standardized questionnaires, physical examinations, interferon-gamma release assays, and chest radiography. All the participants with suspected TB based on the clinical symptoms or abnormal chest radiography results, as well as those with LTBI, were referred for diagnostic investigation in accordance with the national guidelines. Those with an initial diagnosis of TB were then excluded, whereas those with LTBI were included in a follow-up at baseline. Incident patients with active TB were identified from the Chinese Tuberculosis Management Information System, and a multivariate Cox regression model was used to estimate the incidence and risk of TB among those with LTBI.

**Results:**

In total, 667 participants with LTBI were followed up for 1,315.3 person-years, revealing a disease density of 1,292.5 individuals/100,000 person-years (17/1,315.3). For those with LTBI, chest radiograph abnormalities had adjusted hazard ratios for active TB of 4.9 (1.6–15.3).

**Conclusions:**

The presence of abnormal chest radiography findings increased the risk of active TB among older individuals with LTBI in high-epidemic sites in eastern China.

## 1 Introduction

Latent tuberculosis infection (LTBI), a tuberculosis (TB) reservoir, is a continuous source of new patients, with some groups at increased risk of disease onset due to LTBI (Ma et al., 2023). Studies have shown that 5%–10% of people with LTBI develop active TB (Comstock et al., 1974; Vynnycky and Fine, 1997). According to the World Health Organization (WHO) TB report, a quarter of the world’s population is infected with *Mycobacterium* TB (MTB), with approximately 2 billion estimated LTBI cases (World Health Organization, 2022). China is one of the countries with the high LTBI burden worldwide, with an estimated 20% of the people infected with MTB (Institute of Pathogenic Biology et al., 2021). The rate of LTBI trends upward with age (Xiang-wei et al., 2017), reaching 31.38%–44.85% among individuals aged ≥ 60 years (Gao et al., 2015; Tao et al., 2020). As China’s population continues to age, the proportion of older adults has increased from 8.9% in 2011 to 13.5% in 2021(Office of the Leading Group for the Seventh National Population Census of the State Council of China, 2022), a value that exceeds the global average of 9.0% (United Nations Population Report, 2019). Due to decreased immune function, older patients with pulmonary TB often experience other underlying diseases. Furthermore, the clinical manifestations are not typical, and the imaging features are complex. Therefore, pulmonary TB in older individuals is characterized by high incidence, high prevalence, and high mortality (Society of Tuberculosis of Chinese Medical Association, 2023). The severity of pulmonary TB in older individuals should arouse the interest of professional physicians. The older population has gradually become one of the groups most greatly burdened by LTBI and TB. In 2018, a high-level meeting of the United Nations General Assembly on TB called for the acceleration of LTBI screening and preventive treatment to reduce the incidence of active TB caused by LTBI (United Nations high-level meeting on TB, 2018; World Health Organization, 2018).

Zhejiang Province, an economically prosperous province in southeastern China, has a relatively low TB rate in the country. However, previous studies have shown that Quzhou City has the highest incidence of TB in Zhejiang Province, especially in the groups of older individuals over 65 years (Kui et al., 2020; Luo et al., 2024). Two years ago, an LTBI investigation with an interferon-gamma release assay (IGRA) also revealed a high prevalence of LTBI among an older population in Quzhou (Wang et al., 2022). The reported incidence rate of active TB (per 100,000) was 67 in the whole population and 194 in those aged ≥ 65 years, with 41% of patients with TB aged ≥ 65 years in 2019 (Ping et al., 2023). It is necessary to explore the risk factors of TB onset in older individuals with LTBI and take appropriate intervention measures to reduce the incidence of TB in high epidemic sites like Quzhou. LTBI is a high-risk factor for active TB, suggesting that they may be effective targets for preventive intervention; however, the characteristics of the LTBI population that require priority intervention are poorly understood.

To further address these questions, we conducted an LTBI investigation among older individuals in a rural, high-incidence area and followed up individuals with LTBI as a cohort to identify subsequent active TB. We evaluated factors that were likely to influence the progression to active TB among the LTBI cohort during 2 years of follow-up. This research is also the continuation of a previously published study on the burden and predictors of LTBI among older individuals (Wang et al., 2022).

## 2 Methods

### 2.1 Study design and participants

We conducted a cross-sectional study in July 2021 to assess the LTBI status in Quzhou. According to the sampling calculation method and the inclusion criteria in our previous study (Wang et al., 2022), we recruited about 2,000 people and established the LTBI cohort for follow-up. We applied a multistage sampling method. Firstly, we randomly selected two counties (Changshan and Jiangshan Counties) out of six counties in Quzhou City, and then selected the township with the highest TB incidence rate in each of the two counties. The selected two towns were Zhaoxian in Changshan County and Tanshi in Jiangshan County. We then randomly selected three administrative villages in each town and finally determined Xianghu, Gutianfan, and Wuli Villages in Zhaoxian Town, Changshan City and Wang, Tanbian, and Zhan Villages in Tanshi Town, Jiangshan City, as the research sites to derive the sample size. According to the reported data in the TB management information system in China, in the last 5 years, the annual average reported incidence of TB in older individuals (per 100,000) was 273 cases in Changshan County (295 cases in Zhaoxian town) and 192 cases in Jiangshan County (363 cases in Tanshi town), which were much higher than the average provincial level.

### 2.2 Investigation and follow-up

All participants underwent assessments using standardized questionnaires, physical examinations, IGRA, and chest radiography. IGRAs, venous heparin anticoagulant blood, 5 mL, was collected and sent to the county Center for Disease Control and Prevention laboratory under normal temperature, within 4 h. The Quanti FERON-TB Gold In-Tube enzyme-linked immunosorbent assay (CFDA20183400233) was used in one county, and the enzyme-linked immunospot (CFDA20183400233) was used in the other. The determination of test results and quality control were carried out in strict compliance with the kit instructions. All participants underwent chest imaging examinations at the township hospital. Subsequently, the images were uploaded to the picture archiving and communication system of the radiology department of the high-level hospitals for reading by senior doctors. All participants with suspected TB based on clinical symptoms or abnormal chest radiography results, as well as those with LTBI, were referred to the TB-designated hospitals for diagnosis in accordance with national TB diagnostic guidelines to exclude those with an initial diagnosis of active TB. Individuals with LTBI were then included in the follow-up.

Follow-up was from 1 August 2021 to 31 July 2023 and was conducted quarterly by trained community health professionals through on-site visits or wireless phone interviews. During the follow-up period extending from July to August 2022 and from July to August 2023, participants were invited to participate in a period survey, which included suspected symptom inquiry, digital chest photography, and an IGRA. A 2-year close-out visit was scheduled for all participants unless they declined follow-up or died. New cases of active TB were confirmed by doctors in designated TB medical institutions and registered in the TB management information system. None of the individuals with LTBI or abnormal chest radiographs had received any treatment or prophylaxis.

### 2.3 Definition

An abnormality on chest radiography was defined as a partial disease when the clinician ruled out the possibility of active pulmonary TB. Patients with IGRA-positive test findings without clinical evidence of active TB were identified as having LTBI. The LTBI diagnosis was made by professional medical staff. A previous TB infection was determined based on a self-reported history of prior TB disease. Negative IGRA results were considered non-LTBI. According to the “Diagnosis Criterion of Pulmonary Tuberculosis in China (WS288-2017)” (Nation Health and Family Planning Commission of the people’s Republic of China, 2017), pathogenic positivity could be indicated by smear positivity, culture positivity, molecular biology positivity, or pathological positivity. Standard-course TB treatment was provided for all persons diagnosed with TB. Diagnostic investigations included clinical evaluation, sputum smear, sputum culture, GeneXpert test, and chest computed tomography.

The study protocol was approved by the Ethics Committee of the Institute of Quzhou City Center for Disease Control and Prevention. Written informed consent was obtained from all study participants.

### 2.4 Statistical analysis

Microsoft Excel 2007 was used to input and sort the data. IBM SPSS Statistics for Windows, version 19.0 (IBM Corp., Armonk, NY, USA) was used for the statistical analysis. Countable data were described in terms of “number of cases,” “composition ratio (%),” and “rate (%).” A Cox regression model was used to assess the incidence density and risk of TB in the older population. Variables with a *p*-value < 0.1 in the univariate regression analysis were included in a Cox multivariate regression analysis. The incidence density was described as new cases per 100,000 person-years, and the incidence risk was described using an adjusted hazard ratio (95% confidence interval [CI]). Values of *p* < 0.05 were considered statistically significant.

## 3 Results

### 3.1 Baseline characteristics of participants

In 2021, 2,050 permanent residents of rural communities aged ≥ 65 years were considered eligible for the baseline survey, with 1,903 residents completing a physical examination and IGRA test. Among them, seven residents were excluded due to a diagnosis of active TB. Among the remaining 1,896 participants, 667 were IGRA-positive, accounting for 35.2% (667/1,896), and 1,229 were IGRA-negative, accounting for 64.8% (1,229/1,896). IGRA-positive patients were included in the follow-up. At baseline, the proportion of men (57.9% [386/667]) was higher than that of women (42.1% [281/667]). By age group, there was a higher proportion of those aged 65–74 years (66.6% [444/667]) than of those aged ≥ 75 years (33.4% [223/667]). Additionally, there was a higher number of those with an abnormal chest radiograph (40.5% [267/660]) than of those with previous TB (3.4% [23/667]) (**Table 1**).

**Table 1:** Baseline characteristics of participants.

Characteristic	Men [*N* (%)]	Women [*N* (%)]
All participants	386 (57.9)	281 (42.1)
Average age (years)	73.34	73.15
Age group (years)		
65–74	126 (56.3)	98 (43.7)
70–74	126 (57.3)	94 (42.7)
75–79	68 (57.6)	50 (42.4)
≥ 80	66 (62.9)	39 (37.1)
Education level		
Primary school or below	303 (52.9)	270 (47.1)
Junior middle school or above	83 (88.3)	11 (11.7)
Marital status		
Married/cohabiting	331 (61.8)	205 (38.2)
Widowed/divorced/separated/other	55 (42.0)	76 (58.0)
Median body mass index (kg/m^2^)		
Underweight (< 18.5)	37 (62.7)	22 (37.3)
Normal (18.5–24.9)	257 (62.2)	156 (37.8)
Overweight (≥ 25.0)	92 (47.2)	103 (52.8)
Fasting plasma glucose^a^ (mmol/L)		
< 3.9	15 (68.2)	7 (31.8)
3.9–6.0	328 (58.7)	227 (41.3)
≥ 6.0	38 (48.7)	40 (51.3)
Smoking		
Current	155 (99.4)	1 (0.6)
Former/never	231 (45.2)	280 (54.8)
Alcohol use		
Current	194 (92.4)	16 (7.6)
Former/never	192 (42.0)	265 (58.0)
Chest radiograph^b^		
Abnormal	160 (59.9)	107 (40.1)
Normal	221 (56.2)	172 (43.8)
Previous tuberculosis infection		
Yes	15 (65.2)	8 (34.8)
No	371 (57.6)	273 (42.4)

a Fasting plasma glucose results are unknown for 17 participants, 10 men, and 7 women.

b Chest radiograph findings are unknown for seven participants (five men and two women).

### 3.2 Risk of TB among different groups with LTBI

In total, 667 participants with LTBI were followed up for a total duration of 1,315.3 person-years. During the follow-up period, 17 persons with pulmonary TB were identified and diagnosed, and the disease density was 1,292.5/100,000 person-years (17 cases/1,315.3 person-years). During follow-up 19 participants died. One patient was diagnosed with pulmonary TB and died after treatment, whereas the remaining 18 died of other diseases. Multivariable Cox regression showed that, compared with those who showed no abnormalities on chest radiography, the hazard risk ratio of TB in those with chest radiograph abnormalities was 4.898 (95% CI: 1.569–15.290). Other factors, such as sex, age group, median body index, fasting plasma glucose level, smoking status, alcohol use, and previous TB, did not significantly affect the development of active TB (**Table 2**).

**Table 2:** Risk of tuberculosis among different groups with latent tuberculosis infection.

Characteristic	Participants (N)	Person-years	Tuberculosis (*N*)	Incidence rate (% person-year)	Univariable Cox regression		Multivariable Cox regression	
					HR (95% CI)	*p*-value	HR (95% CI)	*p*-value
All participants	667	1315.3	17	1.29				
Gender								
Women	281	553.2	8	1.45	Reference			
Men	386	762.1	9	1.18	0.8269 (0.3189–2.144)	0.696		
Age group (years)					0.341			
65–69	224	449.6	3	0.67	Reference			
70–74	220	436.1	8	1.83	2.710 (0.719–10.215)	0.141		
75–79	118	218.3	3	1.37	2.095 (0.423–10.380)	0.365		
≥ 80	105	211.3	3	1.42	2.001 (0.404–9.917)	0.395		
Education level								
Primary school or below	573	1131.8	12	1.06	Reference		Reference	
Junior middle school or above	94	183.5	5	2.72	2.581 (0.9093–7.328)	0.075	2.58 (0.8266–8.054)	0.100
Marital status								
Married/cohabiting	536	1,062.9	12	1.13	Reference			
Widowed/divorced/separated/other	131	252.4	5	1.98	1.754 (0.6178–4.978)	0.291		
Median body mass index (kg/m^2^)								
Underweight (< 18.5)	59	112.7	2	1.77	1.192 (0.267–5.329)	0.818		
Normal (18.5–24.9)	413	811.8	12	1.48	Reference			
Overweight (≥ 25.0)	195	390.8	3	0.77	0.521 (0.1466–1.846)	0.312		
Fasting plasma glucose^a^ (mmol/L)								
< 3.9	22	44.7	0	0.00	0			
3.9–6.0	550	1,083.8	14	1.29	Reference			
≥ 6.0	78	155.4	1	0.64	0.504 (0.06627–3.833)	0.508		
Smoking								
Current	156	309.8	2	0.65	0.4397 (0.1005–1.923)	0.275		
Former/never	511	1005.5	15	1.49	Reference			
Alcohol use								
Current	208	416.3	4	0.96	0.6726 (0.2193–2.063)	0.488		
Former/never	459	899.0	13	1.45	Reference			
Chest radiograph^b^								
Abnormal	267	513.9	12	2.34	4.523 (1.458–14.02)	0.009	4.898 (1.569–15.290)	0.006
Normal	393	789.7	4	0.51	Reference		Reference	
Previous tuberculosis infection								
Yes	23	42.3	1	2.36	1.865 (0.2473–14.07)	0.545		
No	644	1273.0	16	1.26	Reference			

HR, hazard ratio; CI, confidence interval.

a Fasting plasma glucose results of 17 participants are unknown.

b Chest radiograph findings of seven participants are unknown.

### 3.3 Risk of TB among different periods with LTBI

During the 2-year follow-up, 17 cases developed into active pulmonary TB, of which 64.7% (11/17) developed into active pulmonary TB during the first year and 35.3% (6/17) developed into active pulmonary TB during the second year, with incidence densities of 1,620.7/100,000 and 942.5/100,000, respectively, as shown in **Table 3**.

**Table 3:** Risk of tuberculosis among different periods with latent tuberculosis infection.

Years	Participants (*N*)	Tuberculosis [*N* (%)]	Person-years	Incidence rate (per 100,000 person-years)	95% confidence interval
Cumulative follow-up of 1 year	667	11 (64.7)	678.7	1,620.7	662.41–2,578.99
Cumulative follow-up of 2 years	667	17 (100.0)	1,315.3	1,292.5	779.26–2,106.56

Cumulative follow-up of 1 year is from 1 August 2021 to 31 July 2022. Cumulative follow-up of 2 years is from 1 August 2021 to 31 July 2023.

## 4 Discussion

In 2021, we found that among the older population living in rural areas of Zhejiang province with a high-epidemic prevalence of TB, risk factors for LTBI included the following: male sex, smoking, not opening windows for ventilation frequently at home, and an abnormal chest radiograph. Individuals with these risk factors had an increased risk of developing active TB in LTBI compared with non-LTBI (Wang et al., 2022). However, the risk of progression of active TB among different population groups is not similar; the current research focused on how to identify the high-risk population from LTBI. Our follow-up results showed that individuals with abnormal chest radiographs had an increased risk of progression from LTBI to active TB. Another study showed that in patients with LTBI aged 50–70 years living in rural areas of China, the risk of TB among those with inactive TB lesions on chest radiography was 6.77 times higher than that in the population with a normal chest radiograph (Gao et al., 2017). Those results indicate that this population may be important to target for LTBI screening and preventive interventions in rural areas with a high TB prevalence in China. However, those factors traditionally influencing TB incidence, such as sex, fasting plasma glucose level, smoking status, alcohol use, median body index, and previous TB, did not affect the risk of active TB in individuals with LTBI, especially among patients with a history of pulmonary TB. Previous studies have shown that in those with LTBI, a history of pulmonary TB is a risk factor for the development of active TB (Xin et al., 2020).

Previous studies have shown that the probability of progression from latent TB infection to active TB is 5%–10% (Comstock et al., 1974; Vynnycky and Fine, 1997) in their lifetime, with most developing within 6–24 months after infection (Behr et al., 2018; Li and Gao, 2021). Koul et al. reported a greater probability of active TB development within 2 years of LTBI detection by 5% (Koul et al., 2011). Our study findings showed that the risk of developing active TB within 2 years among older patients with LTBI was 1.29% (17 cases/1,315.3 person-years). This study finding is similar to that reported by Gao et al., who showed that the probability of developing active TB among tuberculin skin test (TST)-/ QuantiFERON-TB Gold In-Tube (QFT-GIT)+ individuals aged > 60 years was 1.45% (Gao et al., 2018). The results showed that 65% of active TB cases occurred within 1 year of LTBI diagnosis, whereas, 35% occurred in the second year.

In 2020, the WHO-issued Comprehensive Guidelines indicated that people with inactive TB are at an increased risk of developing active disease and comprise a target population for post-exposure intervention (World Health Organization, 2020). Guidelines for LTBI management in the USA suggest that people with pulmonary fibrosis lesions are ideal targets for MTB infection detection and preventive treatment (The American Thoracic Association and Centers for Disease Control and Prevention, 2000). With the acceleration of population aging in China, the incidence of TB is increasing in older adults, especially among those with comorbidities, making the standardization of treatment difficult. It is particularly important to identify an ideal target population for preventive treatment in older adults because early detection of active disease has the potential to improve disease management. Carrying out LTBI and chest imaging screening is recommended among older adults in rural areas with a high prevalence of TB in China. To help end the TB epidemic, preventive treatment should be provided for LTBI with abnormal lung imaging findings.

This study has some limitations. First, our study included a small number of investigators and observed new cases. The sites included in the study had a high-transmission background, and the group considered was limited to individuals aged ≥ 65 years; therefore, the risk factors identified are not necessarily applicable to other regions or populations. Second, the 2-year follow-up period to assess disease progression is limited. Individually reported incidence rates may be unstable over time.

In conclusion, this prospective study of active TB among the older population with LTBI tracked the development of active TB in individuals with LTBI. Our results showed that individuals with LTBI still carry a high risk of developing active TB within 2 years. High-risk groups and those with abnormal chest radiographs in rural areas had a high TB prevalence in China. Targeting this population for preventive interventions may improve the chances of ending the TB epidemic.

## Data availability statement

The original contributions presented in the study are included in the article/supplementary material. Further inquiries can be directed to the corresponding author.

## Ethics statement

The studies involving humans were approved by Ethics Committee of Quzhou Center for Disease Control and Prevention. The studies were conducted in accordance with the local legislation and institutional requirements. Written informed consent for participation was not required from the participants or the participants’ legal guardians/next of kin in accordance with the national legislation and institutional requirements. The manuscript presents research on animals that do not require ethical approval for their study.

## Author contributions

PZ: Writing – review & editing, Writing – original draft, Conceptualization, Data curation, Formal analysis, Funding acquisition, Investigation, Methodology, Project administration, Resources, Software. XH: Writing – review & editing, Data curation, Methodology, Validation. WW (3rd author): Conceptualization, Formal analysis, Investigation, Software, Supervision, Writing – review & editing. WW (4th author): Writing – review & editing. BX: Writing – review & editing. BZ: Writing – review & editing. CF: Writing – review & editing. YZ: Writing – review & editing. YG: Writing – review & editing. BC: Conception, Writing – review & editing, Writing – original draft.

## References

[B1] Behr M. A. Edelrtein P. H. Ramalrishnan L. (2018). Revisiting the timetable of tuberculosis. BMJ 362, k2738. doi: 10.1136/bmj.k2738 30139910 PMC6105930

[B2] Comstock G. W. Livesay V. T. Woolpert S. F. (1974). The prognosis of a positive tuberculin reaction in childhood and adolescence. Am. J. Epidemiol. 99, 131–138. doi: 10.1093/oxfordjournals.aje.a121593 4810628

[B3] Gao L. Li X. Liu J. Wang X. Lu W. Bai L.. (2017). Incidence of active tuberculosis in individuals with latent tuberculosis infection in rural China: Follow-up results of a population-based, multicentre, prospective cohort study. Lancet Infect. Dis. 17, 1053–1061. doi: 10.1016/S1473-3099(17)30402-4 28716677

[B4] Gao L. Lu W. Bai L. Wang X. Xu J. Catanzaro A.. (2015). Latent tuberculosis infection in rural China: Baseline results of a population-based, multicentre, prospective cohort study. Lancet Infect. Dis. 15, 310–319. doi: 10.1016/S1473-3099(14)71085-0 25681063

[B5] Gao L. Zhang H. Xin H. Liu J. Pan S. Li X.. (2018). Short-course regimens of rifapentine plus isoniazid to treat latent tuberculosis infection in older Chinese patients: A randomised controlled study. Eur. Respir. J. 52, 1801470. doi: 10.1183/13993003.01470-2018 30361241

[B6] Institute of Pathogenic Biology Chinese Academy of Medical Sciences Chinese Center for Disease Control and Prevention Institute of Geographic Sciences and Natural Resources Research Chinese Academy of Sciences (2021). National expert consensus on estimation of latent infection rate of Mycobacterium tuberculosis. Chin. J. Antitubercul. 44, 4–8. doi: 10.19982/j.issn.1000-6621.20210662

[B7] Koul A. Arnoult E. Lounis N. Andreas K. (2011). The challenge of new drug discovery for tuberculosis. Nature 469, 483–490. doi: 10.1038/nature09657 21270886

[B8] Kui L. Tao L. Vongpradith A. Wang F. Peng Y. Wang W.. (2020). Identification and prediction of tuberculosis in eastern China: Analyses from 10-year population based notification data in Zhejiang Province, China. Sci. Rep. 10, 7425. doi: 10.1038/s41598-020-64387-5 32367050 PMC7198485

[B9] Li M. Gao Q. (2021). Stages of the natural history of tuberculosis: current status and prospects. China J. Antituberc. 43, 1125–1129. doi: 10.3969/j.issn1000-6621.2021.11.005

[B10] Luo D. Wang L. Zhang M. (2024). Spatial spillover effect of environmental factors on the tuberculosis occurrence among the elderly: a surveillance analysis for nearly a dozen years in eastern China. BMC Public Health 24, 209. doi: 10.1186/s12889-024-17644-5 38233763 PMC10795419

[B11] Ma Y. Lu W. Cheng S. M. (2023). Current status of latent infection of Mycobacterium tuberculosis and its prevention and control strategy. Med. J. Chin. PLA. 48, 634–642. doi: 10.11855/j.issn.0577-7402.2023.06.0634

[B12] Nation Health and Family Planning Commission of the people’s Republic of China (2017). WS 288-2017 diagnosis of tuberculosis (Beijing: Standards Press of China).

[B13] Office of the Leading Group for the Seventh National Population Census of the State Council of China. (2022). Bulletin of the Seventh National Population Census (No. 5) - Age Composition of the Population (Beijing, China: National Bureau of Statistics, Office of the Leading Group for the Seventh National Population Census of the State Council). Available at: https://www.stats.gov.cn/tjgb/rkpcjb/qgrkpcgb/202102/t20210628-1818824.html.

[B14] Ping Z. Bingjun X. Chunfu F. Xiaogang H. Wei W. Xing Z. (2023). Analysis on effect of active screening of pulmonary tuberculosis in the elderly in rural area in Quzhou, Zhejiang. Dis. Surveill. 38, 1–6. doi: 10.3784/jbjc.202207180327

[B15] Society of Tuberculosis of Chinese Medical Association (2023). Expert consensus on diagnosis and treatment of pulmonary tuberculosis in the elderly. Chin. J. Tuberc Respir. Dis. 46, 1068–1084. doi: 10.3760/cma.j.cn112147-20230921-00182 37914419

[B16] Tao Z. H. Guangxing Z. H. Yuzheng F. A. Shengbing L. I. Nansen Y. A. Yao H. U.. (2020). Latent tuberculosis infection and its influencing factors among high-risk population in Nanshan District, Shenzhen. China Trop. Med. 20, 702–709. doi: 10.13604/j.cnki.46-1064/r.2020.08.03

[B17] The American Thoracic Association and Centers for Disease Control and Prevention. (2000). Targeted tuberculin testing and treatment of latent tuberculosis infection. This official statement of the American Thoracic Society was adopted by the ATS Board of Directors, July 1999. This is a Joint statement of the American Thoracic Society (ATS) and the Centers for Disease Control and Prevention (CDC). This statement was endorsed by the Council of the Infections Diseases Society of America (IDSA), September 1999, and the sections of this statement. Am. J. Respir. Crit. Care Med. 161, s221–s247. doi: 10.1164/ajrccm.161.supplement_3.ats600 10764341

[B18] United Nations Population Report. (2019). World population ageing 2019 (New York, NY: United Nations). Available at: http://www.un.org/development/desa/pd/zh/node/3172.

[B19] United Nations high-level meeting on TB. (2018). WHO calls for urgent action to end TB: Editors notes: About the UN High-level Meeting on TB The United Nations General Assembly High-level (New York, NY: World Health Organization). Available at: https://www.who.int/news/item/18-09-2018-who-calls-forurgent-action-to-end-tb.

[B20] Vynnycky E. Fine P. E. (1997). The natural history of tuberculosis: The implications of age-dependent risks of disease and the role of reinfection. Epidemiol. Infect. 119, 183–201. doi: 10.1017/S0950268897007917 9363017 PMC2808840

[B21] Wang W. Chen X. Chen S. Zhang M. Hao X. Liu K.. (2022). The burden and predictors of latent tuberculosis infection among elder adults in high epidemic rural area of tuberculosis in Zhejiang, China. Front. Cell Infect. Microbiol. 12. doi: 10.3389/fcimb.2022.990197 PMC9646974 36389154

[B22] World Health Organization. (2018). Latent tuberculosis infection: updated and consolidated guidelines for programmatic management. (Geneva: World Health Organization). Available at: https://www.who.int/publications/i/item/9789241550239. 30277688

[B23] World Health Organization. (2020). WHO consolidated guidelines on tuberculosis: Module 1: Prevention: Tuberculosis preventive treatment (Geneva, Switzerland: World Health Organization). Available at: https://www.who.int/publications/i/item/9789240001503.

[B24] World Health Organization (2022) Global tuberculosis report 2022. Available online at: https://www.who.int/teams/global-tuberculosis-programme/tb-reports/global-tuberculosis-report-2022.

[B25] Xiang-wei L. I. Qi J. I. Lei G. A. O. (2017). A short review on the epidemiology of latent tuberculosis infection in China. Electron. J. Emerg. Infect. Dis. 2, 146–150. doi: 10.19871/j.cnki.xfcrbzz.2017.03.005

[B26] Xin H. Zhang H. Yang S. Liu J. Lu W. Bai L.. (2020). 5-Year follow-up of active tuberculosis development from latent infection in rural China. Clin. Infect. Dis. 70, 947–950. doi: 10.1093/cid/ciz581 31253988

